# How can health be further integrated in urban development policymaking in the United Kingdom? A systems mapping approach

**DOI:** 10.1186/s12961-025-01379-9

**Published:** 2025-07-29

**Authors:** Geoff Bates, Pablo Newberry, Rachael McClatchey, Jack Newman, Sarah Ayres

**Affiliations:** 1 Institute for Policy Research, University of Bath, Bath, United Kingdom; 2 School of Civil, Aerospace and Design Engineering, University of Bristol, Bristol, United Kingdom; 3 School for Policy Studies, University of Bristol, Bristol, United Kingdom; 4 Office for Health Improvement and Disparities, Department of Health and Social Care, Bristol, United Kingdom

**Keywords:** Government, Systems, Health, Prevention, Urban, Cities, Health in all policies

## Abstract

**Background:**

In the United Kingdom the government’s new health mission aims to reduce the burden on healthcare services by shifting from treating poor health to prevention. Delivering this requires action on health in policy arenas outside of the health sector such as urban development, as urban environments are key health determinants. However, change is challenging in complex and long-established policy systems and structures. Systems methods can enhance research into such contexts and demonstrate opportunities for delivering cross-cutting preventative health agendas.

**Methods:**

This study aimed to enhance understanding of how health is integrated in urban development policymaking, and how to bring about change to support healthier development. It was undertaken over two stages. Firstly, a thematic analysis of data from interviews with 37 United Kingdom policy officials exploring urban development decision-making in central government. Secondly, the development of a causal loop diagram based on the variables and connections between them, identified in the interview data.

**Results:**

Analysis revealed how health is not well integrated in urban development policymaking. Through mapping 15 important influencing variables, we identified four main areas where change can be delivered: senior leadership on preventative health, responsibility in urban development teams for health, opportunities in urban development for health experts to promote ideas, and the capacity and capability of officials to act. Addressing any of the factors identified will likely have benefits, but it is by bringing change to multiple highlighted areas that health integration will be maximized.

**Conclusions:**

If the United Kingdom Government’s health mission is to be effective, policymakers must be empowered and incentivized to act on health in areas such as urban development. There is recent evidence of enhanced leadership on health prevention, but this must be supported in in several ways simultaneously, with increased funding, facilitating joined up working across sectors, and enhancing the use of tools and evidence to understand and promote health outcomes. By taking a systems approach this study adds value to existing understandings by going beyond isolated challenges and opportunities, to illustrate the connections between them and, therefore, how any changes are likely to have wider effects.

**Supplementary Information:**

The online version contains supplementary material available at 10.1186/s12961-025-01379-9.

## Background

Globally, non-communicable diseases (NCDs) account for 74% of all deaths [1], but these are largely preventable through appropriate prevention and management strategies of the interacting environmental, behavioural and genetic risk factors [2]. Sustainable Development Goal 3.4 is to reduce premature mortality from NCDs by one-third by 2030 [3]. Many of the risk factors for NCDs are associated with the quality and design of urban environments. A growing body of evidence demonstrates how urban environments have significant health impacts through a range of factors such as housing quality, air pollution, the accessibility and quality of green spaces, noise, neighbourhood walkability and cyclability, and the food environment [4–10]. All of these can be modified by urban development policies and strategies to reduce the burden of NCDs.

For the potential health benefits of urban development policies to be realized, the health effects must be taken into account in both policy development and implementation [4, 11, 12]. Consequently, the World Health Organization’s research priorities for urban health globally include the need to not only continue to build the evidence base on the relationship between urban environments and health outcomes, but to explore how to integrate health into urban agendas and policymaking processes [13]. This article therefore seeks to identify opportunities to influence the uptake of health ideas and evidence in urban development policy in the United Kingdom. There are many influential stakeholders, and urban development outcomes are not outcomes solely of policy and regulations set by central government. For example, market forces dominate the United Kingdom’s housing sector, with most new homes built by private sector volume housebuilders [14], and local authorities in England produce local plans and frameworks to shape development in their area. The agendas, values, capabilities and practices of a wide range of stakeholders will therefore influence the potential to create healthy environments. However, in the United Kingdom, the flow of funding and accountability mean that national government plays an important role in setting the policy context for urban planning decisions at the city- and local-levels [15, 16]. And yet, a recent review of housing and transport policy documents highlighted that health ideas, outcomes and evidence are largely absent in United Kingdom government plans and strategies [17]. While this article primarily focuses on England, it is also important to note the role of devolved administrations in the Scotland, Wales and Northern Ireland. There are examples of initiatives being implemented at the devolved and local level across the United Kingdom that can support health and wellbeing outcomes, such as 15-min cities, low traffic neighbourhoods and reduced traffic speed limits [18–20]. However, given the variation in governance arrangements across the United Kingdom, these policies form a fragmented patchwork that is often disjointed from national strategies [15]. In all these contexts, health is frequently peripheral in policymaking processes and urban planning is not currently widely supporting the conditions for good health [21, 22].

This article responds to this issue by reporting the findings from a study that sought to enhance understanding on how to further integrate health in urban policymaking in the government of the United Kingdom. It seeks to demonstrate how taking a systems approach to researching this policy arena can improve understanding of the factors shaping decision-making, and the multiple interventions that are needed to bring about change to support healthier urban development. The overall research question was: “What are the opportunities to influence the uptake of health ideas and evidence in urban development policymaking?” This is a very timely issue in the United Kingdom, where the new government that came to power in July 2024 has set an urban development agenda based around large-scale and rapid housebuilding, brownfield regeneration and the development of new towns [23]. At the same time, the government has committed to an ambitious new “health mission” including an explicit intention to influence the “wider determinants of health” such as urban environments [24]. The Prime Minister recently called for three big reform shifts to deliver this mission, including from “sickness to prevention”, to prevent people becoming ill [25]. If ideas of prevention and public health outcomes can be effectively integrated into this new urban development agenda it presents an opportunity to create the living conditions to help to reduce the burden of NCDs, which account for 80% of deaths in the United Kingdom [26].

Change in this area remains a significant challenge and there is no guarantee that the health mission will contribute to healthier urban development. There are numerous barriers to a joined-up approach to prioritizing health outcomes in central government in the United Kingdom (Whitehall). It is characterized by its segmented nature, where silos and departmentalism can prevent the effective collaboration needed to tackle complex problems [27, 28]. The government’s urban development policy is shaped by policy objectives in numerous departments. The Treasury is highly influential through its control over public spending, provision of funding and strategic directives, for example through its recent 10 year strategy to improve national infrastructure [29]. Individual departments each have responsibility for different aspects of urban development. For example, responsibility for access to services and facilities is with the Ministry of Housing, Communities and Local Government (MHCLG), highways and active travel with the Department for Transport (DfT) and waste management with the Department of Environment, Food and Rural Affairs (Defra) [30]. Within the Department of Health & Social Care (DHSC), the “Planning and Environments for Health” team has a remit for ensuring that the design of the built environment improves public health [31]. However, outside of DHSC, there has been a lack of incentives for departments to prevent NCDs or improve public health and they instead primarily focus on delivering other objectives through urban development, particularly that of economic growth and productivity [32, 33]. This has been further exacerbated by the post-2010 austerity agenda, which led to particularly deep cuts to strategic capacity and non-essential services, which have disproportionately impacted preventative health programmes. At the same time, public health has faced institutional churn, with creation and abolition of Public Health England created within a decade, and its replacement, the Office for Health Improvement and Disparities, lacking budgetary independence from DHSC.

To maximize the potential for healthier urban development, research can explore how to integrate health further into policy processes. Research into such complex policymaking environments can be enhanced through utilizing systems approaches. Complex adaptive systems are characterized by inter-dependency and feedback between components that influence one another to reinforce or stabilize outcomes [34]. For example, obesity at the population level may emerge as a result of the complex interplay between the prevalence of convenience food, market demand for convenience food, the cost and availability of ingredients, and food policy and legislation [34]. Systems thinking draws upon a range of theories (e.g. general system theory, complexity theory, cybernetics, and learning organizations theory) and methods (e.g. agent-based modelling, network analysis, and system dynamics modelling) to understand complex systems [35], focusing on the interactions between their constituent parts and identifying root causes of problems and opportunities for change [36]. For instance, it can inform preventative health policy by illustrating how contextual factors and implementation processes interact to influence downstream impact [37]. Public health research emphasizes the need for systems approaches, focusing predominantly on the use of methods and tools to investigate complex systems and yield new insights beyond what more traditional reductionist approaches are capable of [38].

Causal loop diagrams are a systems thinking tool that originates from system dynamics, founded by Forrester [39] to guide qualitative analysis and discussion around feedback effects [40]. They have increasingly been used in public health to understand complexity, identify leverage points, design interventions, and inform policy and practice [41, 42]. Furthermore, evidence shows that systems mapping in public health is valuable for generating novel insights, building relationships, and shifting perspectives in the policy process, but it can be challenging to translate the results into changes in policy and practice [43]. Moreover, the United Kingdom Government promotes and provides guidance for civil servants to use systems thinking tools in policy analysis and development [44], and has developed causal loop diagrams to inform strategies for net zero [45] and tackling obesity [46]. In relation to urban health specifically, causal loop diagrams have been used in several ways. This includes demonstrating the influence of urban health indicator tools on urban policy and decision-making [47], understanding the feedback loops influencing health consideration in urban development decision-making from a real estate perspective to inform interventions [48], and investigating the relationships between housing, energy and wellbeing to provide insights for policy design [49].

This article presents a causal loop diagram to visualize how health is integrated in United Kingdom urban policymaking based on the collective views of senior policy officials. It discusses opportunities for change based on key feedback loops within the system that can be leverage points for driving more desirable health outcomes through United Kingdom urban policymaking in central government. In this way, it departs from several dominant strands of public policy analysis in the United Kingdom. First, in contrast to political analysis of individual-decision makers and key office holders (for example this analysis by Byrne [50]), this paper focuses on the systemic nature of decision-making. Second, rather than engage with the constitutional underpinnings and political processes of the Westminster system [51, 52], we analyze the immediate capacities of policymakers to solve a specific policy problem. Third, rather than understanding policymaking as a linear cycle [53], the system map developed in this article seeks to highlight the non-linear complexity and feedback loops of the policy process. This research therefore primarily contributes to theories of policy systems and policymaking complexity, which remains an underdeveloped field, especially in the study of United Kingdom politics and public policy [54, 55]. Crucially, by mapping the complexity of decision-making – without reduction to simplified linear cycles – the paper offers insights into the longstanding challenge of how to embed prevention in public policy [33, 56–58].

## Methods

### Study design

This article describes a study guided by a systems approach that formed part of a large-team transdisciplinary qualitative investigation into how health is included in decision-making processes in the United Kingdom’s urban development system [22]. A detailed description of the methodology is described elsewhere [59]. The article reports the findings of an analysis carried out in two stages. Firstly, a thematic analysis of qualitative data based on interviews with senior policy officials to identify factors influencing the extent that health evidence and ideas are included in urban development decision-making. Secondly, the development of a causal loop diagram based on this analysis of interview data to understand and present this data through a systems lens, and to identify opportunities for change.

### Causal loop diagrams

Causal loop diagrams are conceptual models that represent complex problems by illustrating causal relationships between variables and feedback structures within a system [40, 60]. They can be constructed from any source of information, including interview data and participatory systems mapping workshops [61]. Hence, they are an effective means to externalize mental models and assumptions [62]. In addition, causal loop diagrams can communicate and translate various actors’ perceptions of the system into “useable ideas” [63] and facilitate “focused speculation” on intervention strategies [62]. However, without quantitative data to run model simulations, the behaviour of a causal loop diagram can only be inferred through its structure, which is subject to the perspectives of those involved [64–66]. Recent scoping reviews demonstrate the wide application of causal loop diagrams in public health research [67] and in the context of NCDs and risk factors [43]. Of the 23 studies reviewed by Baugh Littlejohns and colleagues [67], 10 used interview data to develop the causal loop diagrams.

To read a causal loop diagram, a positive causal connection (arrow marked with a “ + ”) indicates a change in the *same* direction (i.e. an increase in variable A causes an increase in variable B, or a decrease in variable A causes a decrease in variable B). A negative causal connection (arrow marked with a “−”) indicates a change in the *opposite* direction (i.e. an increase in variable A causes a decrease in variable B, or vice versa). Causal connections can link together to form feedback loops. Reinforcing feedback loops are virtuous or vicious cycles that cause exponential growth or decline, such as economic growth, climate change and soil erosion. Balancing feedback loops are stabilizing or self-correcting to counteract change and restore and maintain balance, such as environmental regulations that curb pollution and natural processes of carbon dioxide absorption. It is critical to examine feedback loops because not only do they influence the behaviour of systems but they are also potential leverage points for change [36].

### Setting and boundaries of the “system”

This study explores the factors influencing how health is integrated in decision-making that shapes urban development within the central United Kingdom Government. Therefore, the boundaries of the “system” represented in the causal loop diagram and the subsequent discussion of interventions and opportunities for change are that of Whitehall – the factors influencing civil servants and officials who are developing, influencing and implementing policies. As such, the causal loop diagram reflects how different aspects of the problem are perceived and valued by the various stakeholders interviewed, forming a system boundary that inherently limits understanding and opportunities for leveraging change [68]. In addition, we recognize that factors outside of this system are also significant. For example, politics, ideological drivers, corporate interests, the media and public opinion will all influence the direction of Whitehall policy, and urban development outcomes are ultimately shaped by many stakeholders across public and private sectors, all with their own agendas and priorities. While we recognize in our analysis how many of these external factors are influencing the factors in the causal loop diagram, they fall outside of the scope of this work to explore in terms of possible opportunities for change.

### Participants and data collection

A purposive sample was identified to include officials with expertise and experience of urban development decision-making in Whitehall. Participants were identified through a desk-based scoping exercise of relevant government departments and partner organizations. This was supplemented by snowball sampling with professional contacts of the researchers and our interviewees. We prioritized interviewees on the basis of a subjective assessment of their level of expertise and influence in the urban development system, and to include expertise relating to a breadth of roles and departments.

The final sample included senior civil servants and government advisers in government departments and their related non-departmental public bodies and executive agencies with a remit for housing, transport, planning, environment, business, economics and public health. Additional perspectives from policy actors working to influence government policymakers were sought from leaders within currently influential think tanks, policy advisers and officials working at the interface between national and local authorities such as in membership organizations.

Semi-structured interviews with 37 participants were carried out online between May and September 2021. The interviews sought to capture perspectives on the extent to which health is part of decision-making shaping urban development in Whitehall. They included questions on how decisions are made, the extent that officials collaborate with others including health officials, departmental agendas and priorities, the use of evidence, and where ideas come from. The interview guide is provided (see Additional file 1). Informed consent was sought from all interviewees to participate and for interviews to be recorded.

### Analysis stage 1: coding and summarizing interview data

All interviews were transcribed and uploaded into NVIVO 12. All transcripts were reviewed by the researcher who carried out the interview and anonymized so that participants could not be identified. A mixed inductive and deductive coding process was followed [69]. An initial codebook was developed on the basis of key concepts in the study research and interview questions. This was supplemented with new inductive codes added during coding of all interview transcripts using NVIVO 12. Codes were grouped within higher-level categories, such as “political considerations”, “institutions”, “governance”, “legal considerations”, “power and influence”, “evidence”, “actor networks“ and “characteristics of the system”. Written summaries of each category in the codebook were developed, linked with relevant extracts of data, to help the team discuss their shared understandings about the data. This formed the basis for the development of the causal loop diagram.

### Analysis stage 2: development of the causal loop diagram

The process for building the causal loop diagram in this research was based on the steps described by Newberry and Carhart [70] for using interview data analysis as the basis for building the model, and. based on Kim and Andersen’s method [71] for the identification of variables and causal relationships from text data. The steps included:

1. Identification of variables and causal relationships in the summary of interview data analysis
2. Aggregation of microstructures
3. Collaborative and iterative development of causal loop diagram
4. Structure-verification test including supporting interview quotes

Step 1 was carried out by the “model builder”: a researcher with expertise in systems thinking and building causal loop diagrams. Steps 2 and 3 were conducted by the “model builder” and two researchers with expertise in public health, healthy environments and national policymaking in the United Kingdom, one of whom conducted and analyzed the interviews. Step 4 included an additional review of the causal loop diagram by two researchers with expertise in public administration and Whitehall decision-making. The researchers involved are all authors on this paper.

In step 1, variables and causal relationships were elicited from the summary of interview data analysis to form microstructures. These were identified by examining the text for explicit (stated) relationships and implicit (unstated) relationships that could be inferred [70]. Table 1 presents an example of a microstructure identified from a piece of text in the interview data analysis.

**Table 1:** Example microstructure linked to interview data analysis

Text: “The lack of responsibility for health outside the Department of Health and Social Care (DHSC) was linked to the siloed nature of government work”			
Microstructure	Cause variable	Effect variable	Relationship
Microstructure 1	Responsibility for health outside DHSC	Siloed working in national government	Negative/inverse (i.e. change in the opposite direction)

In step 2, the microstructures were aggregated to produce an initial draft of the causal loop diagram. This was achieved by merging variables that were the same or closely aligned while retaining the causal links (e.g. “joined up working on health across departments” and “integration & collaboration on health across national government” were merged into one variable named “joined up working on health”) and inferring additional causal relationships.

In step 3, the causal loop diagram was developed iteratively on the basis of the researchers’ expertise and understanding of the data. For each iteration, the researchers tested the integrity, logic and continuity of the model, revised terminology, moved or removed variables and arrows as necessary, and added variables and interconnections based on the data [72]. Each iteration was checked back against the interview data and each variable in the final map was linked to supporting extracts (see Additional file 2: Supporting data). In this step, there was a significant focus on clarifying feedback loops to help explain why particular system behaviours persist and indicate opportunities for change [36].

To validate the causal loop diagram at step 4, it was subjected to the “structure-verification test” [73]. This included: (a) reviewing the model by people knowledgeable about corresponding parts of the system (i.e. experts in the workings of United Kingdom National Government and policymaking); (b) characterizing variables and/or causal relationships using direct quotes from interviewees, and where necessary; (c) comparing model assumptions to descriptions in relevant literature.

The reporting of this study followed the Standards for Reporting Qualitative Research checklist (see Additional file 3: SRQR checklist).

## Results

**Fig. 1:**
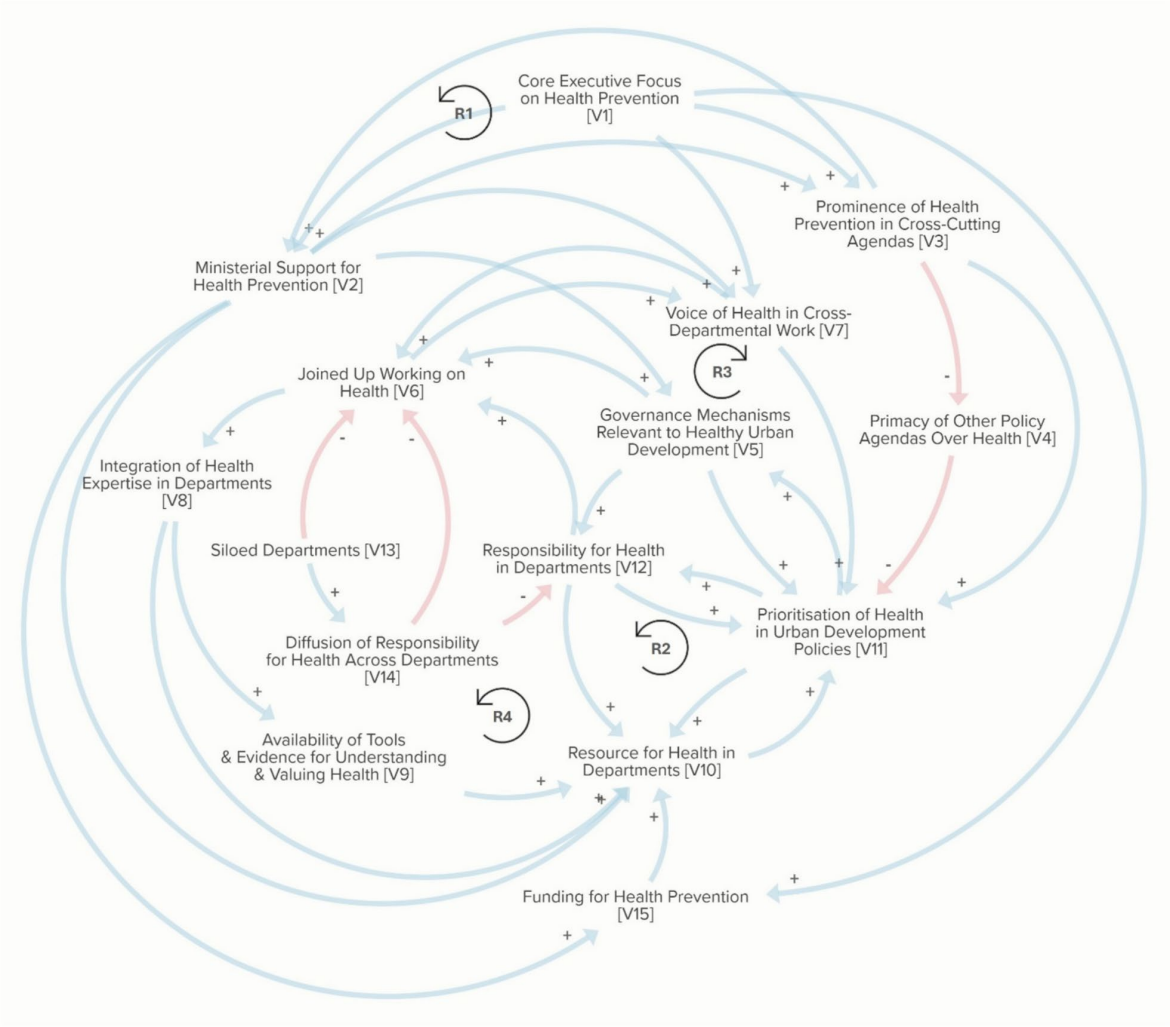
Causal loop diagram representing the integration of health in Whitehall urban development decision-making

The analysis of interview data led to the identification of 15 key variables, described in Table 2. The causal loop diagram (Fig. 1) locates these variables in a system of factors influencing how health is included in Whitehall urban development decision-making. Currently, the 15 variables are not operating in a way that is fully supportive of delivering healthy places. An overarching finding that sits across the variables was that health is currently not well integrated into decision-making, and preventative health is not a key consideration when developing the policies that shape urban development. This is represented by the variable “prioritisation of health in urban development policies” which links with many other variables in the diagram, with health deprioritized through the interaction of these variables. To understand the potential opportunities for shifting this system towards the further integration of health in decision-making, we proceed to discuss the variables and the relationships between them in four reinforcing feedback loops in the diagram that are perceived to have the most influence on the system (R1–R4 in Fig. 1 and Table 3). There were no balancing feedback loops in the diagram. However, there are variables linked to reinforcing feedback loops that may counteract or inhibit their behaviour (e.g. V4, V13 and V14 in Fig. 1).

**Table 2:** Variables influencing the integration of health in Whitehall urban development decision-making

	Variable	Description
V1	Core executive focus on health prevention	The extent that critical actors in government including in the Prime Minister’s Office, Cabinet Office and HM Treasury promote and prioritize preventative health agendas
V2	Ministerial support for health prevention	The extent that ministers and other senior officials in departments with a remit for urban development are supportive of preventative health agendas
V3	Prominence of health prevention in cross-cutting agendas	The extent that health prevention is prioritized in cross-cutting agendas that influence policy setting and objectives across departments
V4	Primacy of other policy agendas over health	The relative weight and influence of departmental or cross-cutting policy agendas over preventative health in goal setting
V5	Governance mechanisms relevant to healthy urban development	Legislation and regulations that shape urban development at all levels in the United Kingdom that include preventative health requirements or conditions
V6	Joined up working on health	The extent that government departments with a remit for urban development or health work together towards shared health objectives
V7	Voice of health in cross-departmental work	The extent to which officials with health expertise and interests are prominent within cross-departmental discussions and policy setting
V8	Integration of health expertise in departments	The extent that health ideas have influence and prominence in decision-making processes within government departments with a remit for urban development
V9	Availability of tools & evidence for understanding and valuing health	Evidence that supports understandings of the links between decisions, health outcomes, and wider societal outcomes, and that can help to make the case for acting on health
V10	Resource for health in departments	The level of capacity and resources to act on health within government departments with an urban development remit
V11	Prioritization of health in urban development policies	The extent that preventative health is prioritized in the policy setting and objectives that shape urban development
V12	Responsibility for health in departments	The extent that departments with a remit for urban development understand preventative health as sitting within their control and responsibilities to deliver on
V13	Siloed departments	The extent that government departments whose policy areas can influence health outcomes work independently and do not engage with one another
V14	Diffusion of responsibility for health across departments	The extent that responsibility for preventative health is spread across government departments and teams
V15	Funding for health prevention	The level of government spending on preventative health activities

**Table 3:** Key feedback loops influencing the integration of health in Whitehall urban development decision-making

Label	Feedback loop	Variables
R1	Senior leadership for preventative health	V2 > V3 > V2
R2	Responsibility for healthier urban development	V12 > V10 > V11 > V5 > V12
R3	Salience of preventative health	V7 > V11 > V12 > V6 > V7
R4	Capacity and capability for health	V6 > V8 > V9 > V10 > V11 > V5 > V6

### Feedback loop 1: senior leadership for preventative health (R1)

The feedback loop highlights the role of senior government actors in facilitating action to improve public health through urban development policymaking. It demonstrates the mutually reinforcing benefits of enhanced support for preventative health from senior politicians and civil servants (V2) and increasing the prominence of this agenda shaping policy across government departments (V3). However, our analysis illustrates how it is likely that influences on the priorities of the “core executive” (the Prime Minister, Cabinet Office and key ministers who are at the centre of policy formation) and of departmental ministers (V1 and V2) falls largely outside of the Whitehall arena, as this feedback loop appears unaffected by changes to other variables.

The priorities of these senior leaders are reflected in the objectives of different departments. When asked about how health sits in their priorities, a civil servant in DfT reflected that, “it’s much more about productivity and average incomes and that type of thing. I think it is in there in the narrative about healthier environments and healthier people, but it comes after the productivity stuff. I’d say that’s probably quite reflective of how the central government thinks”. This extract acknowledges that productivity and growth are established government priorities that influence decisions across departments. Similarly, an official in MHCLG said, “it’s not a responsibility of ours really… our objectives are around delivering economic growth in local areas and empowering places to do that in their own areas… It is not our responsibility to reduce asthma cases”. While the links between urban development and health is recognized, health is rarely included as an important policy objective or outcome by urban department teams with many other priorities to deliver against. For example, when asked if health is a priority in urban development policymaking, an economic adviser in the Treasury replied that, “I don’t think it is. Or at least I don’t think it is explicitly. It’s clear that if you’re in the homelessness team at MHCLG, the health of homeless people is terrible… but if you ask them, is this a health-based policy I don’t think they would say yes”.

Action is therefore driven towards the delivery of other prioritized objectives (V4). Leadership driving health agendas from senior members of the government (V2) and the key departments of the Prime Minister’s Office, Cabinet Office and Treasury (V1) would help to address this. Illustrating this, a parliamentary researcher said that departments were unlikely to be receptive to a greater focus on health outcomes “unless there was a clear lead from something like the Treasury that it was desirable to bring public health matters into policy more widely”. Government action on health outcomes is predominantly focused on health care services, as discussed by a scientific adviser on health, “I think government might prioritize health, but still keep it in the box at the health service and see the health service and the provision of services as the answer”. In contrast, preventative health is not championed from the top: “My impression has always been that it’s [health prevention] one of those policy approaches that nobody will ever stand against; it’s just that nobody will ever stand for it” (Westminster policy analyst).

As illustrated in Fig. 1, if preventative health was championed from senior politicians and civil servants who have the power to shape agendas across government (V1 and V2), the effect would be to raise the profile of this agenda across urban development arenas (V3). This would reduce the primacy of other agendas (V4) that health is currently subordinate to and could lead to changes in legislation and regulations that embed health objectives in decision-making (V5). It would also increase capability to act on health, if ambitions were matched with additional resources (V10) and funding (V15) that would be required to act on a preventative health agenda.

### Feedback loop 2: responsibility for healthier urban development (R2)

Enhancing the priority given to health in urban development policymaking (V11) is therefore one outcome from establishing a stronger preventative health agenda (V3). As illustrated in Fig. 1, this prioritization (V11) is also linked with the variables “responsibility for health” (V12), “governance mechanisms for healthy urban development” (V5) and “resources for health” (V10). Together these form a second feedback loop. A challenge is that health outcomes affect, and are affected by, many areas of urban development policy. Policy areas such as active travel, housing, air quality and the planning system all have clear health implications, but those who are responsible for delivering these policies do not have a remit for health. For example, a DfT official explained how they think about wellbeing outcomes: “I think we recognize it as important – it’s clearly an extremely important aspect of the overall impact on people in communities, but I guess it’s not something that the unit would see as its core mission”. As a scientific advisor in DHSC explained, “the challenge is that trying to address the social determinants of health is very difficult when it falls outside the responsibility of the minister that we are ultimately working for and are accountable to”. For urban development policy officials to address health, responsibility for it needs to be more clearly part of their remit and established as a policy objective in their departments.

Our analysis demonstrates that while one opportunity to promote this comes through feedback loop 1 and a stronger push from the top on preventative health as a cross-cutting agenda (V3), a second opportunity is through the legal and regulatory mechanisms (V5) that embed the need to promote health in urban development policies (V11). Many interviewees highlighted how their statutory responsibilities and the legal requirements they operate under are a factor on their decisions, and how the threat of a challenge by judicial review is an important influence, “it’s [judicial review] certainly one of our criteria around how we work and what we want to achieve” (Environment Agency official). Embedding health outcomes in the regulatory frameworks that urban development teams operate in (V5) would encourage responsibility for this (V12). However, as illustrated in Fig. 1, this in turn is influenced by greater support for preventative health amongst senior leaders (V2) and a greater priority given to health outcomes in urban policymaking (V11).

Our analysis illustrates how if health becomes a greater priority (V11) and responsibility (V12) for urban development officials, they will require greater capacity and resources (V10) to include health in decision-making. Similarly, it would be likely to enhance joined up working between health and urban development teams (V6) to facilitate the knowledge and ideas needed to deliver against health objectives.

### Feedback loop 3: opportunities for promoting health ideas (R3)

This feedback loop highlights the benefits of and challenges for enhancing the voice of health officials (V7) and ideas in urban development decision-making arenas. It demonstrates that enhancing opportunities for health officials to influence the work of urban development teams could increase the extent that health is prioritized in policy setting (V11) through their promoting of the preventative health agenda. Figure 1 illustrates how this requires facilitating greater joined up working between public health and urban development teams (V6) and increasing the relative power of health officials in urban development arenas (V7).

Increasing joined up working between health and urban development officials was a common theme in our interviews, often linked with the need to establish a stronger agenda: “we need a Whitehall narrative on this [incorporating health in urban development]. We need DHSC to be at the same table as MHCLG, the Treasury, Cabinet Office. We need a cross government wide narrative on health and the economy” (official in a national healthcare membership organization). A challenge, however, highlighted by the same official is that the DHSC is not a strong voice in urban development, nor more broadly across government: “One of the reasons health struggles in a Westminster narrative is that it [DHSC] is minimized, as is the ability of the DHSC to set the policy agenda as well as delivering”. Rather than being an influential voice, health officials often must instead support agendas led by other departments. A civil servant in DHSC explained that their department should be setting the agenda on healthy environments in Whitehall but instead compared their role to being akin to “trying to join other people’s parties”.

The impact of urban environments upon health is a complex issue that lands in the portfolios of many departments and teams in these departments. For example, a scientific adviser on the environment explained how different expertise is required to form an effective response to one aspect of urban health, the impact of air pollution, saying it “requires multi-disciplinary input, so we need people who understand the sources and generation of air pollution… We need modelling expertise. We clearly need epidemiologists to be able to interpret the population studies”. Supporting officials to work together and share ideas and expertise is likely to have many benefits, including raising the voice of health officials who are involved (V7). However, effective joined up working (V6) can be negated by siloed departments (V13), which is a characteristic of Whitehall policymaking: “the departmental structure and the nature of splitting ministers by their portfolios… it’s inherently siloed” (cities and local growth unit official). This creates a challenge for joining up on this issue, as summarized by a policy director in a membership organization for local government organizations: “we struggle to give those wicked crosscutting issues the priority they deserve because they are still quite siloed even though we try and work across that”. Consequently, a diffusion of responsibility (V14) plays out where health outcomes are understood as relevant but expected to be taken on elsewhere, “It’s obviously not a case of us not caring about them [health effects], it’s just trusting that they will be picked up by another part of the department” (cities and local growth unit official).

Increasing opportunities for health officials to promote and support action on health in urban development decision-making spaces is therefore a potential area of intervention. Our analysis suggests that giving explicit responsibility over health to urban development teams (V12) may support a positive shift towards more joined up working with health officials (V6). As illustrated in Fig. 1, enhancing the power of health officials would also have the effect of increasing the integration of health expertise (V8), tools and evidence (V9) into urban development departments, enhancing the capacity for healthy policymaking (V10).

### Feedback loop 4: capacity and capability for health (R4)

A challenge for urban development teams to focus on health outcomes is that it requires health expertise (V8) and the capacity and capability to use health data (V8 and V10) to measure and understand wider impacts than their own core objectives. Currently, our analysis illustrates that urban development teams may not seek to do this. For example, an official in DfT said “I guess we wouldn’t normally look at how much it [a policy change] would reduce asthma, for example, but we would very much look at how many tonnes of NO_2_ emissions are you reducing… but we don’t then go onto the next level and look at the outcomes of how many more children are likely to have healthier lungs and so on”. As well as not necessarily having the ambition to look at health outcomes, our analysis demonstrates how a lack of understanding about health and capability to act amongst urban development departments prevents action. An official in DHSC explained that “people like Defra, DfT, are looking at health and wellbeing, they perceive health and wellbeing from their own perspective, it’s not what DHSC believe is public health… there’s just more knowhow within our department that other departments wouldn’t think about”.

The complexity of the policy areas also creates challenges for accounting for health outcomes. This was summarized by an official in Defra: “it’s partly the fact that, when you have environmental benefits, health benefits, congestion benefits and economic benefits – it’s sometimes quite hard to capture all of those and bring them all together in a coherent way”. Several interviewees commented about gaps in evidence and their own knowledge about the complex relationship between urban environments and health: “I think we’re a long way behind on understanding the effect of the built environment” (economic adviser). Improving access to and uptake of evidence and tools (V9) that can be used to understand and demonstrate health benefits is therefore important if officials are to prioritize health outcomes (V11) and act on health. Figure 1 illustrates how this could occur through enhancing contact with health officials through greater joined up working (V6).

However, capacity is also likely to be dependent on greater requirements to focus on health outcomes than at present, which require changes in the other feedback loops discussed here. In particular, greater funding (V15) to enable preventative health to be integrated into urban development processes requires the support of senior leaders (V1 and V2). Limited capacity for integrating health and urban development (V10) is not only an issue for urban development teams, but within DHSC where resources can be limited on this topic: “We may have a commitment on delivering on healthy environments, but we just aren’t putting enough money and resource and manpower into it” (DHSC official). A scientific adviser on the environment linked this challenge with the long-standing priorities of DHSC and the Office of Health Improvement and Disparities being focused on other areas: “I don’t think they had the scientific expertise or maybe even the research budgets to really get to grips with this issue, given all the other issues that they have, like childhood obesity, tobacco smoking, vaping”. Crucially, what is missing is not necessarily the evidence and expertise, but rather the funding and prioritization that would ensure public agencies have the capacity to mobilize existing stocks of knowledge.

## Discussion

This study adds to the literature on how to integrate health in urban development policymaking [4, 74, 75], and specifically in central government [33, 57]. The mechanisms for promoting health outcomes in urban development policymaking identified by our analysis have numerous implications for practice and can help to understand the impact of the government’s “health mission” and renewed focus on prevention., They also have implications for what further action is needed to maximize these potential benefits for creating healthier urban environments. Whilst the findings have originated from interviews with officials in the United Kingdom Government, they have relevance internationally, especially to countries with similar market-led economies and those with similar political systems. We highlight 15 key variables that are currently not operating in a way supportive of healthy urban development. Through the identification of feedback loops, we identify four focal points for shifting this system towards being more supportive to healthy urban development in the United Kingdom: championing of health amongst senior leaders; the responsibility for health outcomes in urban development departments; opportunities for health officials to promote health ideas in urban development processes; and capacity and capability to act on health. All four of these focal points depend on the mobilization of evidence and expertise, but all also emphasize the need for systemic change to ensure existing evidence leads to changes to policy and ultimately to outcomes.

These four areas represent opportunities for change and are consistent with previous literature. In line with Cairney and St Denny [33], we show how “multi-centric policymaking” is a core challenge for preventative approaches, and we build on this by tracing feedback loops that could enable decision centres to precipitate wider systems change. Specifically, by identifying the systemic implications of decisions made by key actors, we show their potential agency to embed preventative health in the policy process [76]. In doing so, we also add to existing research that shows the dysfunctions of key agencies in United Kingdom policymaking, such as the Treasury and Cabinet Office [77, 78], the wider importance of building capacity and establishing effective accountability for a wide range of actors across the system [79, 80]. By applying principles of systems thinking and mapping the factors involved and how they interact, this study adds value to existing understandings of how health is integrated in Whitehall decision-making that often focus on isolated challenges and opportunities. We illustrate the connections between them and, therefore, how any changes are likely to influence the wider system (knock on effects).

Understanding how systems are organized and behave over time is essential to improve their governance in dynamic contexts [81]. However, unless policy and practice shifts from the use of linear frameworks that oversimplify problems to systems approaches that engage with complex realities, major public health challenges will persist [38]. To support practitioners and policymakers in this field, researchers have proposed tools to understand how complex systems function and identify opportunities to leverage change, such as the Action Scales Model [34, 82]. An overarching recommendation is therefore a wider use of systems methods for researching complex policymaking, as this exposes the underlying dynamics involved, and in turn, helps identify key intervention points for policymakers. This can support researchers seeking to comprehend complexity in public policy [55] and for practitioners attempting to deliver preventative policy in the absence of wholesale structural reform [83].

### Implications for further integrating health into urban development

We argue that two of the four areas – leadership and responsibility – are already being enhanced to an extent through the United Kingdom Government’s health mission. The mission and the accompanying rhetoric around health prevention [25] are indications of increased support from senior leaders towards preventative health, though progress remains limited and patchy. By utilizing the systems approach presented here we can demonstrate how additional changes are needed to maximize the opportunities this creates. This is important as attempts from national government to address the wider determinants of health or champion prevention are not new. For example, the previous government’s “levelling up” agenda included improving life expectancy as 1 of 12 missions and used similar rhetoric around shifting to preventative measures [84]. However, the number of people living with NCDs is expected to rise, and health inequalities are expected to widen over the coming years [85]. Additional change is clearly needed beyond agenda setting. Within the context of urban development, the system diagram presented here illustrates how a commitment from the top of government to tackling the wider determinants of health could have far-reaching knock-on effects on Whitehall decision-making. However, to be effective this leadership must go beyond stating the agenda and seek to enshrine health in the priorities, goals and responsibilities of urban development departments (feedback loop R2), and grant health officials a more heightened role in urban development processes (feedback loop R3). In addition, there must be sufficient resources provided to provide teams with the capacity and capability to act (feedback loop R4). Critically, this includes funding. If the promised shift towards prevention is to occur, health funding must be reorganized to reflect this prioritization. Currently around 8% of United Kingdom healthcare expenditure goes towards prevention, compared with nearly two-thirds on treatment [86]. In central government, the health department comprises just 1% of civil servants in core departments [87]. Within this, just 6% of full-time staff work within, and 2% of the budget is spent by, the Public Health and Prevention directorate, with far larger amounts going to the NHS and social care [88].

For healthy urban development, both the levers to create change and the impacts from interventions span multiple departments, making enhanced joined up working across central government a key factor. All the feedback loops would be positively influenced if this was improved. Joined up working across departments is a core aspect of the “mission-oriented approach” [83]. Whilst a full machinery of government change, with responsibility and funding transferred from one department to another by order of the Prime Minister would be challenging to achieve, there are also many smaller, practical changes which can be made to achieve positive steps. For example, data sharing agreements between departments, upscaling collaborations on defined issues with traction (e.g. stronger levers to restrict hot food takeaways near places where young people congregate in the recently revised National Planning Policy Framework [89], or creating space for health and urban development officials to come together around a wider shared agenda in a Health in All Policies approach [90]. Health in All Policies relies heavily on the use of scientific evidence and evaluation tools, such as health impact assessments. These may include city-level quantitative burden of disease, health economic assessments, and citizen and other stakeholders’ involvement to inform the integration of health recommendations in urban policies [74].

This points to another factor that could have knock-on effects by improving capability and capacity to act on health: enhancing access to health evidence and expertise in urban development decision-making processes. It is important to note that this is unlikely to precipitate substantial change on its own, because the prioritization of health over economic pressures will depend on broader systems change, but it is arguably a more achievable area of change in the short-term [76, 91, 92]. Our systems approach illustrates how reaching this lower hanging fruit can support prioritization of health outcomes. It could be achieved by integrating health professionals into urban development teams or decision-making forums, and/or by directly upskilling urban development professionals in health knowledge and skills. As well as through more joined up working, the former might be achieved through health secondments, or dual accreditation/specialist workforce development. Secondments and joint appointments are recommended by the Academy of Medical Science for enhancing organizational relationships [93]. There are growing “healthy place” specialist posts being created within local government, but very limited examples at a national level [94, 95]. The latter could be accomplished through urban development professionals’ curricula, continuing professional development modules, and ensuring ease of access to health data, evidence and tools. There is no curriculum requirement to teach health by the architectural accreditation bodies, and consequently, few architecture courses offer specific health-related content. Whereas in planning, although health is not explicitly mentioned in the curriculum guide [96], there are many hooks which allow the topic to be integrated such as “enhancing the public realm for the benefit of all in society”, and correspondingly, this has a larger emphasis in many curricula and CPD resources [97]. The effectiveness of these various proposed interventions would be further enhanced by strengthening the national body responsible for public health, something that has been weakened in recent years by the austerity agenda and the institutional churn surrounding the creation and abolition of Public Health England.

### Limitations

Our systems approach has added value by enriching our understanding of the interview data and problem space through a model that highlights the key interconnected factors and feedback loops that can be harnessed to reinforce the integration of health in urban development decision-making. However, there are limitations of our approach. Firstly, as this study only focused on factors within central government and not from the wider system, we have not been able to identify influences on all variables. For example, there has been nothing identified from our dataset that influences the Core Executive of the most senior decision-makers in the United Kingdom Government. This highlights the importance of factors outside Whitehall. For example, two recent child deaths linked on death certificates to environmental factors, one to mould, and one to air pollutants, received significant press attention and led to changes in legislation supporting healthier living conditions in Awaab’s and Ella’s laws respectively [98, 99]. International actions have also led to changes, for example in 2021 the Court of Justice of the European Union ruled that the United Kingdom had persistently exceeded nitrogen dioxide levels, leading to the establishment of numerous Clean Air Zones [100]. Similarly, our analysis is limited to the role of the United Kingdom Government *in England*. While many of the findings will also be relevant to the United Kingdom Government’s role in the devolved administrations in Scotland, Wales and Northern Ireland, we do not have the data to draw more comprehensive conclusions.

Secondly, given the causal loop diagram is based on interview data, there was a risk of potential biases and knowledge gaps of participants being reflected in the model [101, 102]. However, this was mitigated by integrating expertise and experiences from 37 participants in a range of positions related to Whitehall urban development decision-making, resulting in a model drawn from their collective views. Furthermore, any potential biases in the researchers’ interpretation of causal connections were reduced through a collaborative and iterative modelling process involving several researchers [70]. We acknowledge though that there may be gaps, and other factors that might enhance the opportunity to integrate health in policymaking could be important. For example, our findings emphasize factors influencing how individuals act and their relationships with others, but in contrast are less focused on opportunities to, for example, develop shared policy targets and budgets across departments. This may reflect the perspectives of interviewees and the scope of the study, but focusing on factors influencing how public health evidence and ideas could create opportunity to change structural factors could be a fruitful area of analysis for future research.

Finally, interview data were relatively weak on some of the variables. For example, with the exception of building safety we found limited examples in our dataset specifically on health in urban development legislation and regulation. Similarly, there were limited quotes relating to the variables of ministerial support (V2) and funding for health prevention (V15). Although these variables were less commonly explicitly discussed in interviews, they were considered essential to the logic of the causal loop diagram, and were retained following review during the validation step. In terms of connections, the link from policy to governance mechanisms was relatively weak but there are examples from practice which reinforce this. For example, “A Fairer Private Rented Sector” White paper led to the Decent Homes Standard refresh (including its extension to Privately Rented Housing) and a Renters’ Rights Bill to legislate making it illegal for landlords or agents to have blanket bans on renting to families with children or those in receipt of benefits [103]. Despite these limitations, this study provides a transparent and data-driven systems analysis in which the model has been developed iteratively and variables and causal connections have been justified through the interview data (see Additional file 2: Supporting data).

## Conclusions

Through taking a systems approach to explore how health can be integrated in urban development decision-making at the national level in the United Kingdom, we identified 15 important influencing variables and, based on the interactions between these variables, four main areas where change can be delivered. By applying these findings to the United Kingdom Government’s health mission, and the current push for a shift towards preventative health, we demonstrate how systems research methods can enhance understandings of policymaking in complex contexts and identify opportunities for delivering cross-cutting preventative health agendas. Key implications from this analysis for the integration of health in urban development is that while leadership to establish the preventative health agenda is a crucial step, to be fully effective it must include elements that establish health outcomes as the responsibility of urban development departments and that enhance the integration of health officials in decision-making processes. It must also seek to enhance the resources necessary to take action on health in urban development departments, which are currently very limited. This includes improving factors such as joined up working between health and urban officials, and the availability of evidence and tools to promote and understand health outcomes, but also critically, to increase funding for health prevention. Addressing any of these factors individually will likely have benefits, but it is by bringing change to multiple areas highlighted in this analysis that the integration of health in urban development policymaking will be maximized.

## Supplementary Information

Additional file 1.

Additional file 2.

Additional file 3.

## Data Availability

Data examples are provided within the supplementary information files. Additional data used and analyzed during the current study are available from the corresponding author upon reasonable request.

## Abbreviations

UK: United Kingdom
NCDs: Non-communicable diseases
MHCLG: Ministry of Housing, Communities and Local Government
DfT: Department for Transport
Defra: Department for Environment, Food and Rural Affairs
DHSC: Department for Health and Social Care
