# Supplementary material for: How can health be further integrated in urban development policymaking in the United Kingdom? A systems mapping approach
Source: Health Res Policy Syst. 2025 Jul 29;23:96. doi: 10.1186/s12961-025-01379-9 (PMC12305967; doi:10.1186/s12961-025-01379-9)
Supplement: Supplementary file 1 — Additional file 1. [file 12961_2025_1379_MOESM1_ESM.docx]

Additional file 1: Interview guide

1. Can you tell us a bit about your role and how it relates to decision-making in urban development?
2. Who are the main actors that you interact with in your work relating to urban development?
3. Where do you think the important decisions are made that influence urban development?
4. How (if at all) do legal considerations influence policymaking?
5. What do you think are the important influences on discussions and decision-making relating to urban development?
6. What dominant ideas or narratives are currently most influential on your organisation's thinking?
7. What are the priorities of your organisation for urban development?
8. To what extent do you think that health and wellbeing is a priority in urban development in your organisation/ institution?
9. How do you think health and wellbeing relates to your department's important policy priorities?
10. What do you think restricts the ability to create healthy places?
11. What are the main forms of evidence that your department/ organisation use when coming to policy decisions? What do you find most and least effective to use?)
12. What do you think could be introduced into the policy development process to support healthier development? For example, evidence, regulations, processes.
13. If you were to consider health and wellbeing in your decision-making, what data would you find most useful or clear?
14. How can public experiences of health and wellbeing impacts be effectively communicated to decision makers?
15. Is there anything that you think we haven't asked you about that would be important for you to tell us?
