# Supplementary material for: How can health be further integrated in urban development policymaking in the United Kingdom? A systems mapping approach
Source: Health Res Policy Syst. 2025 Jul 29;23:96. doi: 10.1186/s12961-025-01379-9 (PMC12305967; doi:10.1186/s12961-025-01379-9)
Supplement: Supplementary file 2 — Additional file 2. [file 12961_2025_1379_MOESM2_ESM.docx]

Appendix 1

**Supporting interview data for the variables in the causal loop diagram**

Each variable in the causal loop diagram (figure 2) is linked with interview data. Two extracts for each variable are provided here as examples of the supporting dataset.

| **#** | **Variable** | **Definition** | **Example supporting quotes** |
| --- | --- | --- | --- |
| V1 | Core Executive Focus on Health Prevention | The extent that critical actors in government including in the Prime Minister's Office, Cabinet Office and HM Treasury promote and prioritise the preventative health agendas. | "I don’t know that the departments would be, unless there was a clear lead from something like the Treasury that this was desirable, that it was desirable to bring public health matters into policy more widely". Civil Servant in a Parliamentary research role (asked if other departments would be receptive to including health outcomes in their work)   "I would say, probably the number 10 policy unit, you feel, is the top target because it can be the engine of getting things done and it has the authority of the Prime Minister and thus if you really want to get departments to look at it, then that’s seen really as the kind of main target" Think Tank Director (asked where power and influence lies) |
| V2 | Ministerial Support for Health Prevention | The extent that Ministers and other senior officials in Whitehall departments with a remit for urban development are supportive of preventative health agendas. | “Have we got enough people shouting about the health aspects? Is there enough pressure? I think it’s quite interesting how it compares or fits in with climate emergency stuff, where that’s really high up on the agenda, quite rightly and then is health part of that narrative or is it separate?” Civil servant, DfT   “My impression has always been that it’s (health prevention) one of those policy approaches that nobody will ever stand against; it’s just that nobody will ever stand for it” Policy Adviser working across government departments |
| V3 | Prominence of Health Prevention in Cross-Cutting Agendas | The extent that health prevention is prioritised in other cross-cutting agendas that influence policy setting and objectives across departments. | “I think government might prioritise health, but still keep it in the box at the health service and see the health service and the provision of services as the answer”. Scientific Advisor on Health  “The Department of Health and Social Care is primarily geared up to treat disease and is not there to prevent disease. Public Health England and its predecessor were – fell more into the area of prevention, but really, they didn’t – I don’t think they had the scientific expertise or maybe even the research budgets to really get to grips with this issue, given all the other issues that they have, like childhood obesity, tobacco smoking, vaping” Scientific Advisor on the Environment |
| V4 | Primacy of Other Policy Agendas Over Health | The relative weight and influence of departmental or cross-cutting policy agendas over preventative health in goal setting. | "So I think when you look at some of the narrative around levelling up you get - it’s much more about productivity and average incomes and that type of thing, rather than – I think it is in there in the narrative about healthier environments and healthier people, but it comes after the productivity stuff. I’d say that’s probably quite reflective of how the central government thinks”. Civil Servant, DfT   “If you were to say, ‘What’s the big agenda?’ it wouldn’t be, ‘We’re gonna really transform mental health,’ or ‘We’re going to really drive down obesity rates.’ They’re often, ‘Let’s improve the natural environment, and really improve our environment’ because of a whole host of reasons, including it improves physical health and mental health, or, ‘Let’s level up so places have more economic activity, more civic engagement,’ and the bi-product of that is better physical and mental health”. Civil Servant, DEFRA |
| V5 | Governance Mechanisms Relevant to Healthy Urban Development | Legislation and regulations that shape urban development in the UK that include preventative health requirements or conditions. | "That [legislation] could be a really powerful policy lever to get action on air quality as well. We, on several occasions have been able to make the case for more resources to deliver or been able to talk to local authorities about more radical measures that are unpopular with local businesses because of the legal requirement". Civil Servant, DEFRA   "They’re [legal requirements] important because they add gravitas to our position to say look, this isn't just to meet a planning policy, this is to meet a wider legal requirement... I mean there’s more scope for wriggle room, could you say, around policy interpretation but certainly around legal requirements there is obviously a greater risk of legal challenge ... So it is very important and we are very aware and use them to best effect" Civil Servant, Environment Agency |
| V6 | Joined Up Working on Health | The extent that government departments with a remit for urban development work or for health work together towards shared health objectives. | “we need a Whitehall narrative on this [incorporating health in urban development]. The funding pots is a good example actually. We need the department of health and social care to be at the same table as MHCLG, the Treasury, Cabinet Office. We need a cross government wide narrative on health and the economy” Official in a national healthcare membership organisation  "the nature of the issue [air pollution and health] that we are dealing with, requires multi-disciplinary input, so we need people who understand the sources and generation of air pollution. We need people who understand the accurate measurement of air pollution or reporting of it. We need modelling expertise. We clearly need epidemiologists to be able to interpret the population studies, which have been so powerful in linking poor air quality and poor health outcomes" Scientific Advisor, environment |
| V7 | Voice of Health in Cross-Departmental Work | The extent to which actors with health expertise and interests are prominent within cross-departmental discussions and policy setting. | “So in a way DHSC should be leading on this agenda, you know, linking up with other departments rather than us looking into other departments, you know, and trying to influence their work. But that is incredibly difficult for the department. Because that’s always reacting to something, it’s more intensive, more frustrating than you leading on a piece of work and others joining you, than you trying to join other people’s parties if you like, yeah”. Civil servant, DHSC   "What I would say about DHSC which is a bit strange so the Lansley reforms ten years ago now almost, they basically moved 95% of the control and the budget of the NHS away from the DHSC to NHS England which is an alternative body. So what’s interesting is the DHSC actually one of the reasons health struggles in a Westminster narrative is that that minimised, and the ability of the DHSC to set the sort of the policy agenda as well as delivering". Official in a national healthcare membership organisation |
| V8 | Integration of Health Expertise in Departments | The extent that health ideas have influence and prominence in decision-making processes within government departments with a remit for urban development. | “One of the reasons health struggles in a Westminster narrative is that it [DHSC] is minimised, as is the ability of the DHSC to set the sort of policy agenda as well as delivering”. Public Health official  *Officer Cities & Local Growth Unit:* “(Health hasn’t) been a significant focus for our work and no we don’t have a health person in each team.  *Interviewer*: "So bringing in that health element relies on you guys kind of picking it up and working with partners to give it some edge?"  *Officer, Cities & Local Growth Unit "*Yes it generally relies on probably very specific drivers and/or interests, and/or opportunity really” |
| V9 | Availability of Tools & Evidence for Understanding and Valuing Health | Evidence that supports actors to understand the links between decisions, health outcomes, and wider societal outcomes, and that can help to make the case for acting on health. | "It’s partly the fact that, when your environmental benefits, health benefits, congestion benefits, and economic benefits – it’s sometimes quite hard to capture all of those, and bring them all together in a coherent way” Civil servant, DEFRA   “I guess we wouldn’t normally look at how much it (a policy change) would reduce asthma, for example, but we would very much look at how many tonnes of NO 2 emissions are you reducing? Those are things that you can put a value on, but we don’t, then, go onto the next level and look at the outcomes of how many more children are likely to have healthier lungs and so on”. Civil servant, DfT |
| V10 | Capacity & Resources for Health in Departments | The level of capacity and resources to act on health within government departments with an urban development remit. | “Obviously we’re not health experts so we can't say designing a development in a certain way is intrinsically more healthy than other way because we don’t have that expertise, that’s a very important specialism” Civil servant, DEFRA   "We may have a commitment on delivering on healthy environments, but we just aren’t putting enough money and resource and manpower into it", Civil Servant, DHSC |
| V11 | Prioritisation of Health in Urban Development Policies | The extent that preventative health is prioritised in the policy setting and objectives that shape urban development. | *Interviewer:* What are the Treasury's priorities for urban development?"  *Economic Adviser, HMT:* “Supply. Period. No ifs. No buts. Supply”.   “It’s all about supply and increasing supply, and we just need to get more houses built... I think that is the central objective of housing policy at the moment – is to increase supply to 300,000 a year, and there are subsets of that around beauty and place-management, but the central commitment in housing policy is, clearly, around housing” Think Tank director |
| V12 | Responsibility for Health in Departments | The extent that departments with a remit for urban development understand preventative health as sitting within their control and responsibilities to deliver on. | “the challenge is that trying to address the social determinants of health is very difficult when it falls outside the responsibility of the minister that we are ultimately working for and are accountable to”. Scientific adviser on health   “I often thought, if I worked in the Department of Transport, what would my thought process be? It would be to decrease congestion. It would be to improve the travelling experience. It would be to make – get more information to people about smart motorway systems, all this sort of stuff. I wouldn’t be thinking about the negative consequences of transport, so therefore I wouldn’t – if someone told me, well what you do every day is responsible for, we think, up to 30,000 deaths, premature deaths in the UK every year, you really should be doing something about it. I think that would be something I would not want to hear, and I’d probably shy away from”. Scientific Advisor, Environment |
| V13 | Siloed Departments | The extent that government departments whose policy areas can influence public health outcomes work independently and do not engage with one another. | ”people just don’t have the responsibility for it (thinking about health outcomes). So people still very much work in silos and do their own little bit…What we need to do is create a sort of grouping that everything has to go through so that health becomes everyone’s responsibility”. BEIS official    "although health inequalities is particularly important right now, we’re tending to use a number of our different teams to work on them rather than creating new resource to reflect its priority..., we struggle to give those wicked crosscutting issues the priority they deserve because they are still quite siloed even though we try and work across that". Policy Adviser working across government departments |
| V14 | Diffusion of Responsibility for Health Across Departments | The extent that responsibility for preventative health is spread across government departments and teams. | “I actually think it’s a very confused situation because we have, historically, we have DEFRA, who have got the responsibility for measuring and reporting air pollution. We have got the Department of Transport in many urban areas and certainly, up until the directly elected mayors, responsible for the regulation of those vehicles and their emissions, so the cause of a lot of the problem. Then the other end of the spectrum, we have got the Department of Health - the Department of Health and Social Care it now is, who actually should be very influential in trying to improve health by talking to those other departments and maybe leading on the policy needs going forward”. Scientific advisor on the environment   It’s obviously not a sort of case of us not caring about them (the health effects of urban development), it’s just the sort of trusting that they will be picked up by another part of the department" Civil Servant in the Cities and Local Growth Unit |
| V15 | Funding for Health Prevention | The level of government spending on preventative health activities. | “looking at what the budget is spent on, and what the focus is, it is still downstream on the treatment of services despite that being totally unsustainable and it’s a bit of an anathema for us. Politicians want sustainability and to spend less money on things, but then don’t actually invest in prevention and the things that will lead to that. Housing being one of them that could save NHS money” Scientific Advisor, health   I think government, if they wanted to really help with these issues, they could provide funding and I’m talking about Treasury here... which would be to the public’s benefit down the line and would be good use of taxpayer’s money ...But politics is short-termism". Scientific Advisor, environment |
