# Supplementary material for: How can health be further integrated in urban development policymaking in the United Kingdom? A systems mapping approach
Source: Health Res Policy Syst. 2025 Jul 29;23:96. doi: 10.1186/s12961-025-01379-9 (PMC12305967; doi:10.1186/s12961-025-01379-9)
Supplement: Supplementary file 3 — Additional file 3. [file 12961_2025_1379_MOESM3_ESM.docx]

Additional file 3: Standards for Reporting Qualitative Research (SRQR)

The SRQR checklist provided here was used to guide the reporting of this study. It is adapted from O'Brien BC, Harris IB, Beckman TJ, Reed DA, Cook DA. 2014. Standards for reporting qualitative research: a synthesis of recommendations. *Academic Medicine*, 89 (9).

|  |  | **Section/ place** |
| --- | --- | --- |
| **Title and abstract** | |  |
|  | **Title** - Concise description of the nature and topic of the study Identifying the study as qualitative or indicating the approach (e.g., ethnography, grounded theory) or data collection methods (e.g., interview, focus group) is recommended | Title provided |
|  | **Abstract** - Summary of key elements of the study using the abstract format of the intended publication; typically includes background, purpose, methods, results, and conclusions | Abstract provided |
|  |  |  |
| **Introduction** | |  |
|  | **Problem formulation** - Description and significance of the problem/phenomenon studied; review of relevant theory and empirical work; problem statement | Background: throughout but specifically para 2, 4, 5) |
|  | **Purpose or research questio**n - Purpose of the study and specific objectives or questions | Background (para 2, 3) |
|  |  |  |
| **Methods** | |  |
|  | **Qualitative approach and research paradigm** - Qualitative approach (e.g., ethnography, grounded theory, case study, phenomenology, narrative research) and guiding theory if appropriate; identifying the research paradigm (e.g., postpositivist, constructivist/ interpretivist) is also recommended | Background (para 5, 6); Methods, subheadings Study design and Causal loop diagrams |
|  | **Researcher characteristics and reflexivity** - Researchers’ characteristics that may influence the research, including personal attributes, qualifications/experience, relationship with participants, assumptions, and/or presuppositions; potential or actual interaction between researchers’ characteristics and the research questions, approach, methods, results, and/or transferability | Methods, subheading Analysis stage 2: development of the causal loop diagram (para 2). |
|  | **Context** - Setting/site and salient contextual factors | Methods, subheading Setting and boundaries of the ‘system’ |
|  | **Sampling strategy** - How and why research participants, documents, or events were selected; criteria for deciding when no further sampling was necessary (e.g., sampling saturation) | Methods, subheading Participants and data collection para 1 |
|  | **Ethical issues pertaining to human subjects** - Documentation of approval by an appropriate ethics review board and participant consent, or explanation for lack thereof; other confidentiality and data security issues | Declarations section |
|  | **Data collection methods** - Types of data collected; details of data collection procedures including (as appropriate) start and stop dates of data collection and analysis, iterative process, triangulation of sources/methods, and modification of procedures in response to evolving study findings; rationale** | Methods, subheadings study design (para 1), participants and data collection (para 1, 2, 3) |
|  | **Data collection instruments and technologies** - Description of instruments (e.g., interview guides, questionnaires) and devices (e.g., audio recorders) used for data collection; if/how the instrument(s) changed over the course of the study | Methods, subheading participants and data collection (para 3) and additional file 1 |
|  | **Units of study** - Number and relevant characteristics of participants, documents, or events included in the study; level of participation (could be reported in results) | Methods, subheading participants and data collection (para 1, 2, 3) |
|  | **Data processing** - Methods for processing data prior to and during analysis, including transcription, data entry, data management and security, verification of data integrity, data coding, and anonymization/de-identification of excerpts | Methods, subheading analysis stage 1: coding and summarizing interview data (para 1) |
|  | **Data analysis** - Process by which inferences, themes, etc., were identified and developed, including the researchers involved in data analysis; usually references a specific paradigm or approach; rationale** | Methods, throughout subheadings analysis stage 1: coding and summarizing interview data, analysis stage 2: development of the causal loop diagram |
|  | **Techniques to enhance trustworthiness** - Techniques to enhance trustworthiness and credibility of data analysis (e.g., member checking, audit trail, triangulation); rationale** | Methods, subheading analysis stage 2: development of the causal loop diagram (para 6) |
|  |  |  |
| **Results/findings** | |  |
|  | **Synthesis and interpretation** - Main findings (e.g., interpretations, inferences, and themes); might include development of a theory or model, or integration with prior research or theory | Findings (para 1 and figure 1) |
|  | **Links to empirical data** - Evidence (e.g., quotes, field notes, text excerpts, photographs) to substantiate analytic findings | Throughout findings section and in additional file 2 |
|  |  |  |
| **Discussion** | |  |
|  | **Integration with prior work, implications, transferability, and contribution(s) to the field -** Short summary of main findings; explanation of how findings and conclusions connect to, support, elaborate on, or challenge conclusions of earlier scholarship; discussion of scope of application/generalizability; identification of unique contribution(s) to scholarship in a discipline or field | Discussion (throughout, but specifically para 1, 2, 3); Conclusion |
|  | **Limitations** - Trustworthiness and limitations of findings | Discussion: subheading limitations |
|  |  |  |
| **Other** | |  |
|  | **Conflicts of interest** - Potential sources of influence or perceived influence on study conduct and conclusions; how these were managed | Conflicts of interest statement provided |
|  | **Funding** - Sources of funding and other support; role of funders in data collection, interpretation, and reporting | Funding statement provided |
|  |  |  |
